# Long-term coral microbial community acclimatization is associated with coral survival in a changing climate

**DOI:** 10.1371/journal.pone.0291503

**Published:** 2023-09-22

**Authors:** James T. Price, Rowan H. McLachlan, Christopher P. Jury, Robert J. Toonen, Michael J. Wilkins, Andréa G. Grottoli

**Affiliations:** 1 School of Earth Sciences, The Ohio State University, Columbus, Ohio, United States of America; 2 Department of Microbiology, Oregon State University, Corvallis, Oregon, United States of America; 3 Hawai‘i Institute of Marine Biology, School of Ocean and Earth Science and Technology, University of Hawai‘i at Mānoa, Honolulu, Hawai‘i, United States of America; 4 Department of Soil and Crop Sciences, Colorado State University, Fort Collins, Colorado, United States of America; Biodiversity Research Center, TAIWAN

## Abstract

The plasticity of some coral-associated microbial communities under stressors like warming and ocean acidification suggests the microbiome has a role in the acclimatization of corals to future ocean conditions. Here, we evaluated the acclimatization potential of coral-associated microbial communities of four Hawaiian coral species (*Porites compressa*, *Porites lobata*, *Montipora capitata*, and *Pocillopora acuta*) over 22-month mesocosm experiment. The corals were exposed to one of four treatments: control, ocean acidification, ocean warming, or combined future ocean conditions. Over the 22-month study, 33–67% of corals died or experienced a loss of most live tissue coverage in the ocean warming and future ocean treatments while only 0–10% died in the ocean acidification and control. Among the survivors, coral-associated microbial communities responded to the chronic future ocean treatment in one of two ways: (1) microbial communities differed between the control and future ocean treatment, suggesting the potential capacity for acclimatization, or (2) microbial communities did not significantly differ between the control and future ocean treatment. The first strategy was observed in both *Porites* species and was associated with higher survivorship compared to *M*. *capitata* and *P*. *acuta* which exhibited the second strategy. Interestingly, the microbial community responses to chronic stressors were independent of coral physiology. These findings indicate acclimatization of microbial communities may confer resilience in some species of corals to chronic warming associated with climate change. However, *M*. *capitata* genets that survived the future ocean treatment hosted significantly different microbial communities from those that died, suggesting the microbial communities of the survivors conferred some resilience. Thus, even among coral species with inflexible microbial communities, some individuals may already be tolerant to future ocean conditions. These findings suggest that coral-associated microbial communities could play an important role in the persistence of some corals and underlie climate change-driven shifts in coral community composition.

## Introduction

Increasing concentrations of atmospheric CO_2_ are leading to global warming and ocean acidification, threatening the long-term survival of corals and the persistence of coral reef ecosystems. By the year 2100, tropical ocean temperatures are expected to rise 1–3°C with a parallel increase in acidity of 25–200% (approximately 0.1–0.3 pH units) [1]. These changing conditions may lead to reduced coral skeletal growth [e.g., 2–4] coupled with increases in coral bleaching [e.g., 5–7], disease outbreaks [e.g., 8–10], and coral mortality [e.g., 6, 11–13]. However, some corals may already possess traits which confer tolerance to these conditions, or they may have the potential to acclimatize to ocean warming and acidification, and these corals are more likely to form the communities that survive this century [14–17]. Recent work by McLachlan et al. [18] supports this potential tolerance, finding that coral species with wider variation in physiological phenotype are more likely to possess the ability to acclimatize.

Corals can acclimatize to potential future ocean conditions through several mechanisms including shuffling to more thermally tolerant Symbiodiniaceae species [e.g., 19–21], maintaining or increasing their energy reserves [e.g., 21–25], increasing the contribution of heterotrophy [e.g., 12, 26–28], and changing gene expression [e.g., 15, 29–31]. Flexibility of the bacterial and archaeal communities of a coral, hereafter referred to as microbial communities, has also been posited as a potential mechanism which may allow corals to acclimatize to a changing climate [e.g., 32–35].

Coral-associated microbial communities play important roles within the coral holobiont (i.e., the coral host, endosymbiotic algae, and microbial communities together), such as influencing disease resistance and nutrient cycling [e.g., 33, 36–38]. A recent field study by Price et al. [39], showed links between the coral holobiont physiology and associated microbial communities, and between environmental conditions and associated microbial communities in some species. More specifically, some coral species shift their microbial community composition when experimentally exposed to warmer waters [e.g., 34, 35, 40, 41], more acidic conditions [42], or both [35, 43]. These changes in microbial community composition have previously been linked to stressed, bleached, or otherwise unhealthy corals [35]. But experimental investigations of the microbial responses to thermal stress have typically lasted only days to weeks [e.g., 34, 35, 44, 45], with the longest experiments lasting between two and six months [43, 46, 47]. These studies provide important information on short to moderate-term (as defined by Grottoli et al. [48]) microbial responses to heat stress or coral bleaching, but it remains unclear if or how microbial responses persist over multi-year periods of stress, such as those expected later this century. It is also unclear if these community shifts confer resistant characteristics to the coral or are simply a sign of degrading health [33].

To determine the potential link between coral-associated microbial communities and coral persistence in the face of chronic global ocean warming and acidification, we characterized microbial community composition of four Hawaiian coral species following a 22-month outdoor mesocosm experiment. Corals were exposed to ocean acidification, ocean warming, and a combined dual stress of ocean warming and acidification treatments representing conditions expected later this century [49, 50]. We hypothesized that shifts in coral-associated microbial communities to future ocean conditions is a strategy related to coral survivorship in response to multi-year chronic treatment. Further, since coral physiology can change dramatically in response to ocean warming and acidification, we hypothesized that the coral-associated microbial community composition correlates with the overall coral physiology. Here we characterize the responses of tropical coral-associated microbial communities to chronic ocean warming and acidification over a multi-year time frame, providing important insight into the potential roles of these microbial communities for the acclimatization and persistence of corals this century.

## Methods

### Experimental design and coral collection

This study was conducted between February 2016 –December 2017 in a mesocosm setup at the Hawai‘i Institute of Marine Biology (HIMB) on Moku O Lo‘e Island (24.43413°N, 157.78802°W), adjacent to the island of O‘ahu, Hawai‘i, USA. The mesocosm setup has been more fully described previously [50–53]. Briefly, forty flow-through mesocosms (0.5 m x 0.5 m x 0.3 m, ~70 L) were divided into a fully factorial design with two pH levels (present day pH of ~8.0 vs. ocean acidification with pH at –0.2 relative to present day levels, ~7.8) and two temperature levels (present day daily average of 23.5–27.5°C vs. ocean warming of +2.0°C above present day), resulting in four treatments (n = 10 mesocosms per treatment) that ran for 22 months with full seasonal and daily variability in light and seawater conditions. The mesocosms were originally stocked with approximately a 2 cm layer of carbonate reef sand and gravel from an adjacent backreef, three 10–20 cm pieces of reef rubble, a juvenile convict surgeonfish (*Acanthurus triostegas*), a threadfin butterflyfish (*Chaetodon auriga*, a generalist grazer of non-coral invertebrates), and ramets from the eight most common reef-building coral species from the Hawaiian archipelago [54, 55]. Seawater was unfiltered and any organisms that entered the mesocosms through the inflow were allowed to remain. Neutral density mesh was used to reduce light levels to be similar to collection depth on the reef.

The four coral species included in this study were *Montipora capitata*, *Porites compressa*, *Porites lobata*, and *Pocillopora acuta*. These corals, selected to be ecologically and phylogenetically diverse, are four of the eight most common species across the Hawaiian Archipelago [54, 55], and represent three of the most common reef-building coral families worldwide (Acroporidae, Poritidae, and Pocilloporidae). Six parent colonies (i.e., genets) from each species were collected between 17 August and 13 November 2015 from a depth of 0.5–5 m at each of four sites (Hale‘iwa, Moku o Lo‘e, Sampan, and Waimānalo) surrounding the island of O‘ahu (S1 Fig, Table 1). The vast majority of corals were collected at a depth of 2 ± 1 m, but small differences in reef geomorphology among sites required a few colonies to be collected from slightly shallower (0.5–1 m at Moku o Lo‘e and Sampan) or deeper (3–5 m at Electric Beach and Hale‘iwa) depths. One of the species, *Porites lobata*, does not naturally occur at Moku o Lo‘e, so it was not sampled at that site. A 5–10 cm ramet (branch or mound) was removed underwater via hammer and chisel from parent colonies separated by at least 5 m to minimize the possibility of sampling clonally derived colonies, as well as to avoid biasing the sampling towards particular micro-environments [56, 57]. Genets were later confirmed by genotyping the colonies using available microsatellite markers [58, 59], and no clones were identified based on identical multilocus genotypes from the same site, suggesting low probability that any were clonally derived. Each genet was then fragmented into four ramets, attached to a ceramic plug, and randomly assigned to one of the 10 mesocosms in each treatment. Coral ramets were acclimated to the mesocosms for at least 3 months under present-day Hawaiian seawater temperature and ambient pH conditions (i.e., similar to the control conditions) prior to the commencement of the experiment. On 1 February 2016 the experiment began with a gradual increase of +0.5°C and a decrease of 0.05 pH units, according to treatment, over a period of 20 days to avoid shocking the mesocosm communities. The final treatment conditions were reached on 20 February 2016 and were as follows: (1) control treatment (mean present day temperature and pH), (2) ocean acidification treatment (present day temperature and –0.2 pH units), (3) ocean warming treatment (+2.0°C and present-day pH), and (4) future ocean treatment (+2.0°C and –0.2 pH units). These corals were maintained in mesocosm conditions for 22 months until sampling between 25 November and 04 December 2017. Full temperature and pH records throughout the study are presented in previous work [50, 51, 53] and full experimental metadata is described in Table 2. Coral ramets that died as well as the full complement of reef-associated organisms which recruited into the mesocosms were retained to ensure that they replicated a reef-like environment. As juvenile fish grew larger their densities were slowly reduced, and they were rotated among mesocosms about every other week to maintain similar levels of algae and invertebrate grazing throughout the experiment.

**Table 1:** Four ramets were collected from six genets of each species at all four sites, except for *Porites lobata* which was collected from three sites. One ramet from each genet was represented once in each treatment at the start of the experiment. Shown is the total number of coral ramets in each treatment at the beginning of the mesocosm experiment, followed by the number of ramets sampled for microbial community analysis at the end of the 22-month experiment in parentheses. The number of sampled ramets was less than the number of initial ramets due to the 30% live tissue coverage mortality threshold after 22 months.

Collection Site	Treatment	*Porites compressa*	*Porites lobata*	*Montipora capitata*	*Pocillopora acuta*
Moku o‘ Loe	Control	6 (6)		6 (6)	6 (4)
21.43417° N,	Acidification	6 (3)		6 (5)	6 (6)
157.78634° W	Warming	6 (4)		6 (2)	6 (3)
	Future Ocean	6 (3)		6 (2)	6 (3)
Sampan Channel	Control	6 (5)	6 (6)	6 (5)	6 (6)
21.45239° N,	Acidification	6 (6)	6 (6)	6 (5)	6 (6)
157.79487° W	Warming	6 (4)	6 (3)	6 (5)	6 (5)
	Future Ocean	6 (4)	6 (3)	6 (5)	6 (3)
Hale‘iwa	Control	6 (5)	6 (5)	6 (3)	6 (6)
21.59252° N,	Acidification	6 (5)	6 (6)	6 (4)	6 (6)
158.11034° W	Warming	6 (1)	6 (4)	6 (0)	6 (1)
	Future Ocean	6 (4)	6 (3)	6 (0)	6 (0)
Waimānalo	Control	6 (5)	6 (6)	6 (6)	6 (6)
21.32629° N,	Acidification	6 (6)	6 (6)	6 (6)	6 (5)
157.67460° W	Warming	6 (3)	6 (4)	6 (1)	6 (0)
	Future Ocean	6 (4)	6 (3)	6 (1)	6 (0)

**Table 2:** Meta-data of experimental methods. Adapted from Grottoli et al. (2021) Table 2. All dates in YYYY-MM-DD format.

CORAL COLLECTION	Latitude and longitude of collection sites	Collection depth	Collection dates	Coral species and morphology	Symbiodiniaceae for all coral species	Acclimation prior to experiment
	Moku o Lo‘e: 21.434167 N, 157.786335 W Waimānalo: 21.326287 N, 157.674599 W Sampan: 21.452394 N, 157.794870 W Hale‘iwa: 21.592516 N, 158.110337 W	Between 0.5 and 5 meters	Between 2015-08-29 and 2015-11-11	*Porites compressa* (branching), *Porites lobata* (massive), *Montipora capitata* (branching and encrusting), *Pocillopora acuta* (Branching)		At least 82 days, between 2015-11-11 and 2016-02-01
EXPERIMENTAL DESIGN	Name of experimental location	Stress treatment period	Tank system type	Number of tanks per treatment	Number of coral genets per treatment	Number of recovery days post-stress
	Hawai‘i Institute of Marine Biology on Moku o Lo‘e in Kāne‘ohe Bay, Hawai‘i, USA	Between 2016-02-20 and 2017-12-13	Flow-through	10 tanks	18 to 24 genets, per species	0 days
EXPERIMENTAL TEMPERATURE AND *p*CO_2_ CONDITIONS	Stress temperature and *p*CO_2_ above MMM	Control temperature and *p*CO_2_	Baseline temperature and *p*CO_2_	Temperature and *p*CO_2_ ramp-up rate	Duration at stress temperature and/or *p*CO_2_ stress level	Temperature and *p*CO_2_ modulation
	+2°C and + 350 μatm	~23.5–27.5°C over the annual cycle and ~400 μatm *p*CO_2_	~23.5–27.5°C over the annual cycle and ~400 μatm *p*CO_2_	0.5°C and 0.05 pH unit increments every 10 days	662 days	diurnally and seasonally varying
OTHER EXPERIMENTAL CONDITIONS	Light conditions & cycle	Flow rate (cm s^-1^)	Tank turnover rate	Seawater source and filtration	Salinity, nutrients, and dissolved oxygen over the annual cycle	Coral feeding:
	Outdoor with 30% shade cloth. Diurnally and seasonally varying	Not measured	Inflow rate was ~1.2 L min^-1^ with a residence time of 1 hour	Natural seawater pumped directly from adjacent reef, unfiltered	Salinity: ~34.47–34.68 psu Nutrients: Dissolved Oxygen:	No supplemental feeding

### Coral mortality

All coral ramets sampled in this study were photographed between 25 November and 10 December 2017, except for approximately half of the *Pocillopora acuta* ramets due to technical issues. Coral mortality at the end of the 22-month experiment was assessed visually through estimations of live tissue coverage from the photographs and direct visual examination of each coral ramet. Coral ramets were divided into those with less than 30% surface coverage of live tissue and those with greater than 30% live tissue coverage. Though this categorization is imperfect, only ramets with greater than 30% live coral tissue were sampled for microbial community composition to avoid incorporating marginal or dead tissue into the analyses. Further, corals with less than 30% live tissue included those that had died or likely would soon (see also Anthony et al. [60] for an example of similar thresholds).

### Coral-associated microbial community sampling

Subsamples (1–3 cm^2^) were collected from the growing tip of each surviving ramet of the three branching coral species across the four treatments using sterile bone cutters and while wearing gloves. For *Porites lobata*, subsamples were removed using a small sterile cork borer due to the mounding morphology of this species. Once a subsample was removed from the ramet, it was immediately placed into a 5 ml Eppendorf tube (Hamburg, Germany) filled with 20% DMSO-0.5 M EDTA saline saturated solution (pH = 8.0) preservative, shipped to The Ohio State University, and stored at room temperature for no more than 1.5 months. A full list of collected subsamples is in Table 1.

At The Ohio State University, each sample was rinsed lightly with autoclaved ultrapure 0.22 μm filtered artificial saltwater (3.5% NaCl) to remove residual preservation buffer. The coral tissue was then removed from the skeleton by airbrushing with sterile artificial seawater. DNA was extracted from the resulting slurry using PowerSoil DNA Isolation kits (Qiagen, Hilden, Germany) following the manufacturer protocol. Successful extraction of genomic DNA was confirmed using a Qubit fluorometer prior to amplification of the V5-V6 region of the 16S rRNA gene using the primers CS1_784F and CS2_1061R (forward: 5’-AGGATTAGATACCCTGGTA-3’; reverse: 5’-CRRCACGAGCTGACGAC-3’). These primers included CS1 and CS2 linkers to allow the downstream application of adapter sequences and sample-specific barcodes. Polymerase chain reaction (PCR) was completed in two stages. Stage one PCR used Amplitaq Gold 360 DNA polymerase (Thermo Fisher Scientific, Waltham, Massachusetts, USA) in 25 μl reaction volumes. Stage one PCR cycling conditions were as follows: 15 min at 95°C, followed by 28 cycles of 95°C for 30 s, 55°C for 30 s and 72°C for 30 s, with a final extension time of 10 min. Successful amplification was visualized via gel electrophoresis. Stage two PCR used MyTaq HS mastermix (Bioline, Memphis, Tennessee, USA) in 20 μl reaction volumes and cycling conditions were as follows: 95°C for 5 minutes, followed by 8 cycles of 95°C for 30 seconds, 60°C for 30 seconds, and 68°C for 30 seconds. A final elongation period was performed at 68°C for 7 minutes. These amplicons were subsequently prepared for multiplexed sequencing on an Illumina MiniSeq sequencer (2 x 153 base pairs, mid-output). The second stage of the PCR process and the Illumina sequencing were completed by the DNA Services Facility at the University of Illinois at Chicago.

Reads produced by Illumina sequencing were processed using the QIIME software package version 1.9 [61]. Within QIIME, forward and reverse reads were joined, filtered at a quality threshold of 20, and adapters and primers removed. Operational taxonomic units (OTUs) were clustered at 97% similarity and taxonomy was assigned based on release 132 of the Silva ribosomal database [62] via UCLUST [63]. Chimeric reads associated with these OTUs were removed via USEARCH [63]. Remaining OTUs were retained only if 10 reads or greater were present across all samples to limit inclusion of errant sequences. Any OTUs which were identified as chloroplast, mitochondria, or eukaryotic in origin were removed from further analyses. A negative PCR control was used to identify laboratory contaminants, but this blank contained a small number of reads and no known laboratory contaminants were identified in this sample. All bacteria classified within the order, Halanaerobiales, were also removed because these bacteria are commonly used in the laboratory where work was completed and are not typically found in the habitat associated with these Hawaiian corals [39]. Prior to diversity analyses, two samples with final read counts below 500 were also removed to limit the consideration of samples with extremely low sequencing depth. All raw, unprocessed reads are available on NCBI’s Sequence Read Archive under accession number PRJNA645714.

### Statistical analyses

All analyses were performed using R software package version 3.5.0 [64] and PRIMER v6 [65]. Statistical significance was defined as α ≤ 0.05. Each coral genet was represented in all four treatments of this experiment, such that the effects of treatment could be assessed independent of genet.

First, to assess whether microbial community composition differed among coral species and coral collection locations at the end of the experiment, both alpha diversity and beta diversity were compared among corals in the control. The control serves as a reference for the baseline microbial community composition of the corals. Alpha diversity of microbial communities among coral species and collection sites was measured using all reads via the number of observed OTUs, Chao1 (estimated species richness) [66], Shannon’s Diversity Index [67], and Faith’s phylogenetic diversity (Faith’s PD) [68]. The phylogeny for Faith’s PD was constructed with representative sequences via fasttree [69]. Alpha diversity values were compared using a Kruskal-Wallis one-way analysis of variance and a post hoc Dunn’s Test. All alpha diversity metrics and subsequent statistical analyses were performed via R package ‘vegan’ (v2.5–7), except Faith’s PD, which used R package ‘picante’ (v1.8.2). A rarefaction curve was also performed for all samples via R package ‘vegan’. Next, beta diversity was calculated with a Bray-Curtis dissimilarity matrix [70] from normalized OTU data and then compared using a permutational analysis of variance (PERMANOVA). The Bray-Curtis dissimilarity matrix was also used in the creation of NMDS plots (via R package ‘vegan’ v2.5–7) to visualize some of the beta diversity comparisons. Similarity percentage analyses (SIMPER) were used to identify the microbial OTUs that differed most in relative abundance among coral species and collection sites and thus were the greatest contributors to dissimilarity between sample groupings.

**Fig 1:**
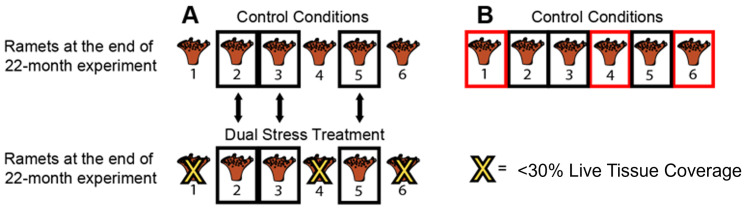
Diagram showing how coral-associated microbial communities were compared within and among treatments, using the future ocean treatment as an example. (A) To evaluate if the microbial community composition changed in response to the future ocean treatment, comparisons were restricted to genets that survived and were able to be sampled in both the control and the future ocean treatment. (B) To evaluate if the baseline microbial community composition differed among genets based on survival in future ocean treatment, comparisons were restricted to genets in the control whose lineages had greater than 30% live tissue coverage (shown in black squares) or less than 30% live tissue coverage (shown in red squares) following 22 months in the future ocean treatment.

Next, the data was analyzed using two approaches to determine if microbial community composition was related to the treatment conditions. First, to assess whether coral-associated microbial communities differed between the control and treatments after 22 months, alpha and beta diversity of each coral species were compared between ramets of genets that survived in both the control and each treatment condition (Fig 1A). Second, to determine whether coral survival in the future ocean treatment was related to coral-associated microbial composition of the different genets, alpha and beta diversity were compared between coral ramets in the control whose ramets of the same genet survived versus those that died in the future ocean treatment (Fig 1B).

We assessed if coral-associated microbial community composition was correlated to the coral physiological profile measured in McLachlan et al [18]. The physiology profile consisted of the following measurements: total biomass, protein, lipid, Symbiodiniaceae density, color, total organic carbon flux (TOC), photosynthesis rates, respiration rates, maximum *Artemia* sp. capture rate, and calcification rate. TOC flux, photosynthesis, respiration, and *Artemia* capture were measured on live coral during the last 20 days of the experimental period (23 November– 13 December 2017), calcification was measured as the integral rate over the entire 22 months, while all other measurements were completed following sacrifice, initial freezing at –20°C, transport to Ohio State University on dry ice, and long term freezing at -80°C. Descriptions of the methods used to measure these physiological characteristics and interpretations of the results are detailed in McLachlan et al. [53]. Physiological measurements were available for *P*. *compressa*, *P*. *lobata*, and *M*. *capitata*. These physiological measurements were considered together for multivariate analyses and are hereafter referred to as the overall physiological profile. The overall physiological profile was compared (1) among coral species in the control and (2) between paired ramets of genets that survived in the control and future ocean treatment via PERMANOVA with 9999 permutations. Next, BEST analyses were used via PRIMER to test for relationships between the coral-associated microbial community composition and overall physiological profile of (1) each coral species in the control and (2) paired ramets of genets that survived in both the control and future ocean treatment. Each BEST analysis used the Spearman rank correlation via the BIOENV method with 99 permutations. The BEST analysis creates correlations between the Bray-Curtis dissimilarity matrix of the microbial data with the Euclidean distance matrix of normalized coral physiological data and identifies which physiological variables best explain the microbial community composition [65].

## Results

**Fig 2:**
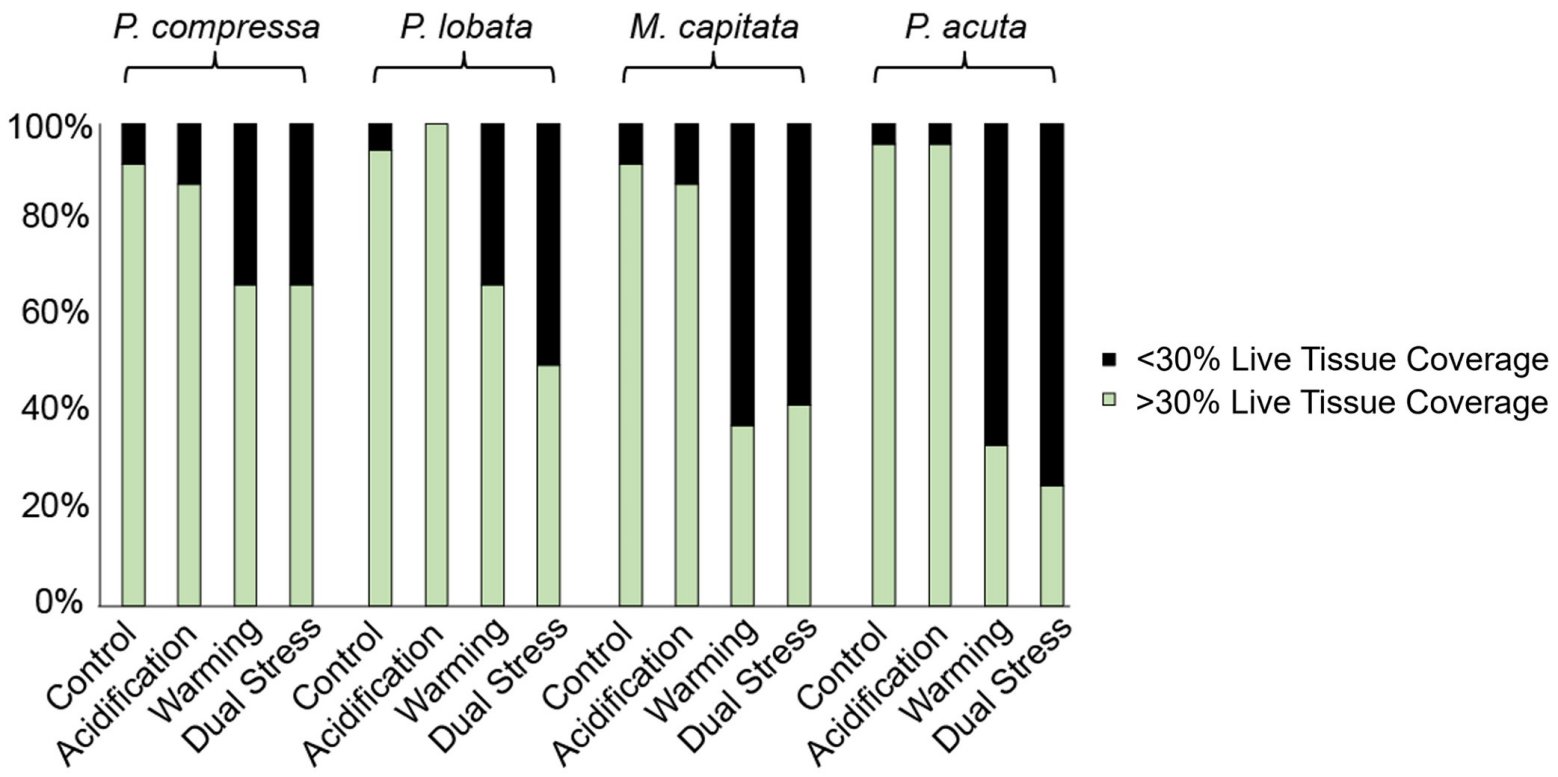
Percent survivorship based on 30% live tissue threshold for each coral species in each treatment at the end of the 22-month mesocosm experiment.

Using the >30% live tissue threshold, average coral survivorship in the control and the ocean acidification treatments was high (both 93%) and substantially lower in the ocean warming (51%) and future ocean treatments (46%). Within species, survivorship in the ocean warming and future ocean treatments was highest in *P*. *compressa* (66% in both treatments) and *P*. *lobata* (66% and 50.0%, respectively), followed by *M*. *capitata* (38% and 42%, respectively), and lowest in *P*. *acuta* (33% and 25%, respectively) (Fig 2).

**Fig 3:**
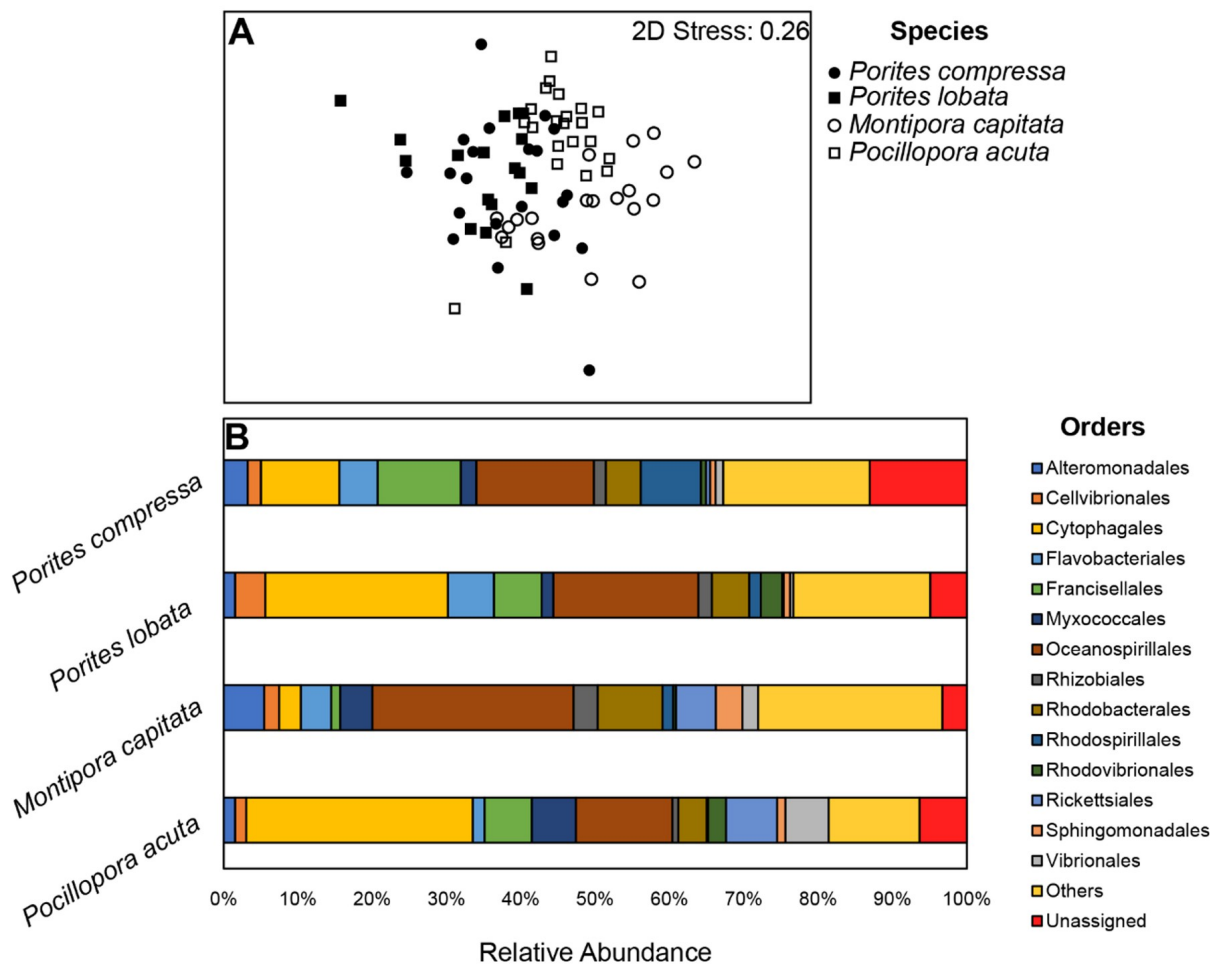
Microbial communities associated with corals in the control. (A) NMDS plot of microbial communities associated with each coral species in the control. Each species significantly differed from the other (See S2a Table in S1 File for PERMANOVA statistical details). (B) Mean Relative abundances of the most common microbial Orders associated with each coral species in the control. Only Orders with a relative abundance greater than 2.0% in at least one coral species are represented individually.

Overall, there were 10,747 OTUs across the 4,755,266 sequences included in this analysis of microbial communities. The samples had a mean read count of 19,896.5 ± 11,938.5 (see rarefaction curve in S2 Fig). Across the four coral species, the most abundant OTUs were associated with the orders Oceanospirillales and Cytophagales, primarily in the genera *Endozoicomonas* and *Candidatus* Amoebophilus, respectively (Fig 3).

### Variation in coral-associated microbial diversity of corals in the control

Within the control samples, beta diversity of the microbial communities, but not alpha diversity, differed among all four coral species, though the two *Porites* species were the least dissimilar (Fig 3A, S1, S2 Tables in S1 File). *M*. *capitata* had the greatest relative abundance of Oceanospirillales (27.1%) while *P*. *acuta* hosted the greatest relative abundance of bacteria in the order Cytophagales at 28.6% (Fig 3B). Within each species in the control, alpha and beta diversity of coral-associated microbial communities did not differ based on the provenance of the ramets for *P*. *compressa* and *P*. *lobata* but did differ based on collection location of some ramets for *M*. *capitata* and *P*. *acuta* (S2-S4 Tables in S1 File). Specifically, *M*. *capitata* and *P*. *acuta* corals originally collected from Hale‘iwa hosted distinct microbial communities from those originally collected from Moku o Lo‘e (see S3D, S3E Table in S1 File) with lower richness in *M*. *capitata* corals from Moku o Lo‘e (S4 Table in S1 File). The microbial communities associated with *M*. *capitata* corals from Hale‘iwa and Waimānalo both had a lower abundance of the order Oceanospirillales in comparison with conspecifics collected from the two Kāne‘ohe Bay sites (Sampan and Moku o Lo‘e), primarily due to a reduced abundance of one OTU (JOKG01000007.7100) from the genus *Endozoicomonas* (S5A Table in S1 File). The difference among sites for microbial communities associated with *P*. *acuta* corals, however, was largely driven by a lower relative abundance of a *Candidatus* Amoebophilus sp. OTU (JQ515688.1.1518) and a higher relative abundance of a Myxococcales OTU at Moku o Lo‘e compared to conspecifics at other sites (S4B Table in S1 File).

The physiological profiles of *P*. *compressa*, *P*. *lobata*, and *M*. *capitata* all differed from each other (S6 Table in S1 File, see McLachlan et al. [53] for detailed discussion of physiological data). However, BEST analysis revealed no significant correlation between the overall physiology of these coral species and their associated microbial communities (S7 Table in S1 File).

### Microbial community comparisons among treatments

**Fig 4:**
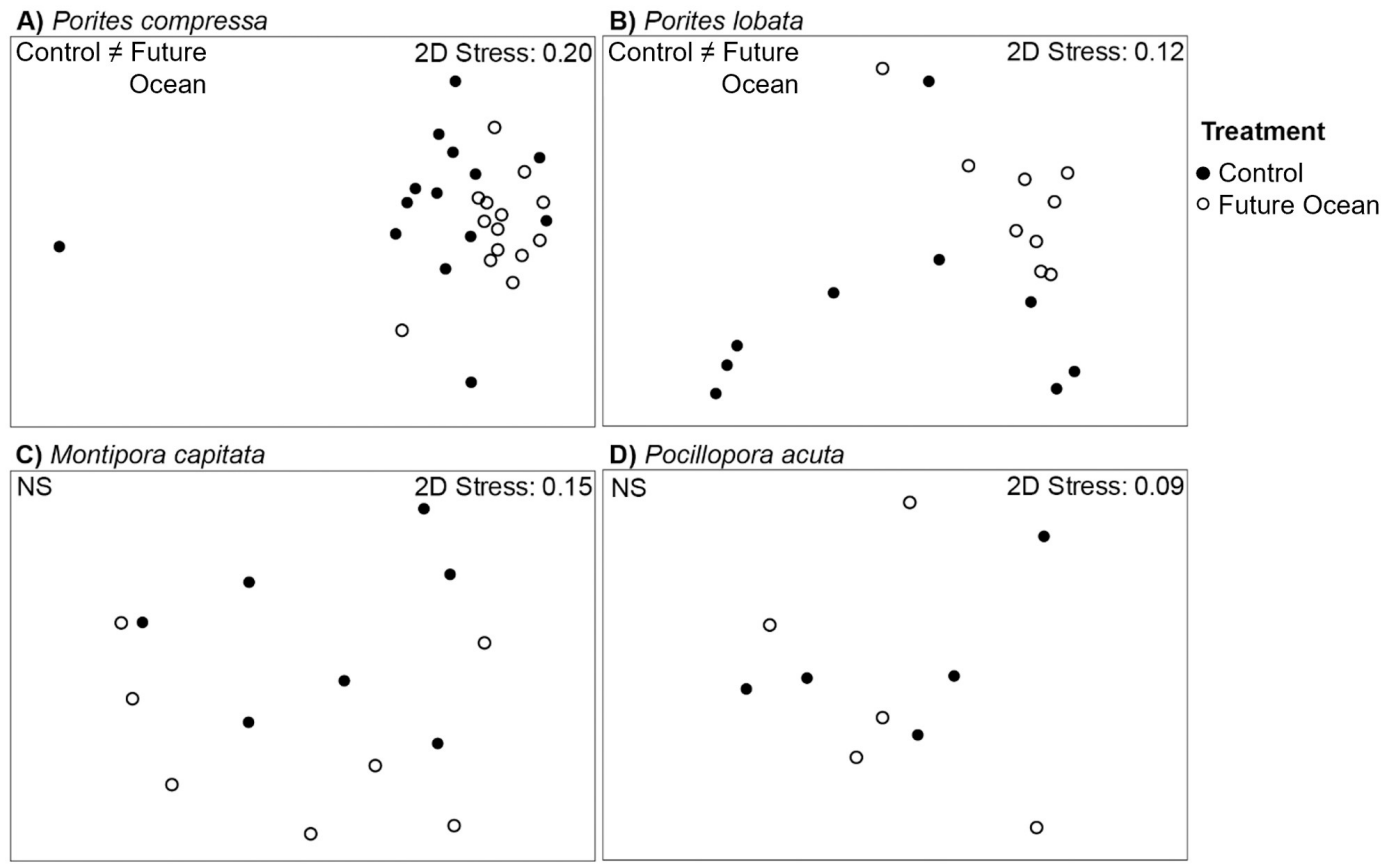
NMDS plots of coral-associated microbial community composition between the control (closed circles) and the future ocean treatments (open circles) for genets of each coral species that survived the future ocean treatment (Illustrated in Fig 1A). Significant differences detected by PERMANOVA analyses (p < 0.05) are indicated in the top left corner (NS = no significant difference) with corresponding statistical details in S7 Table in S1 File. Comparison of microbial community composition by Order between the control and future ocean treatments for each coral species is in S3 Fig.

Among genets that survived, microbial community alpha diversity did not significantly differ between controls and ocean acidification treatment, or controls and ocean warming treatments for any coral species (S8A, S8B Table in S1 File). However, the alpha diversity of microbial communities associated with *Porites lobata* and *Porites compressa* was significantly greater in the control ramets than in the future ocean treatment ramets (S8C Table in S1 File). Ocean acidification also had no effect on the beta diversity of the coral microbial communities (S9A Table in S1 File). However, ocean warming did affect the beta diversity of microbial communities associated with *P*. *lobata* (S9B Table in S1 File). In the future ocean treatment, microbial communities associated with *P*. *compressa* and *P*. *lobata* also differed significantly from the control (S9C Table in S1 File; Fig 4).

For both Porites species, the beta diversity differences were driven by the abundance of one specific OTU in the order Oceanospirillales (MUIA01000001.189.1735; Kistimonas sp.) in the control but near absence in the future ocean treatment (S10A, S10B Table in S1 File). This difference was more pronounced in P. lobata than in P. compressa (S10A, S10B Table in S1 File). Interestingly, *Porites compressa* in the future ocean treatment had an almost three-fold increase in the relative abundance of bacteria of the order Cytophagales, from 5.7% in the control to 14.2% in future ocean conditions, while *Porites lobata* actually had a slight decrease from 22.0% to 17.5% in the same bacterial order (S3 Fig). Changes in relative abundance of Cytophagales for both *Porites* corals was driven primarily by one OTU of the bacterial genus *Candidatus* Amoebophilus (JQ515688.1.1518) (S10A, S10B Table in S1 File).

The overall physiological profile differed between the control and the future ocean treatment only in *P*. *compressa* (S11 Table in S1 File). However, BEST analysis revealed no significant correlations between the overall physiology of any coral species and their associated microbial communities in the control and future ocean treatments (S12 Table in S1 File).

### Microbial community comparisons between coral genets within the control based on fate of their ramets in the future ocean treatment

**Fig 5:**
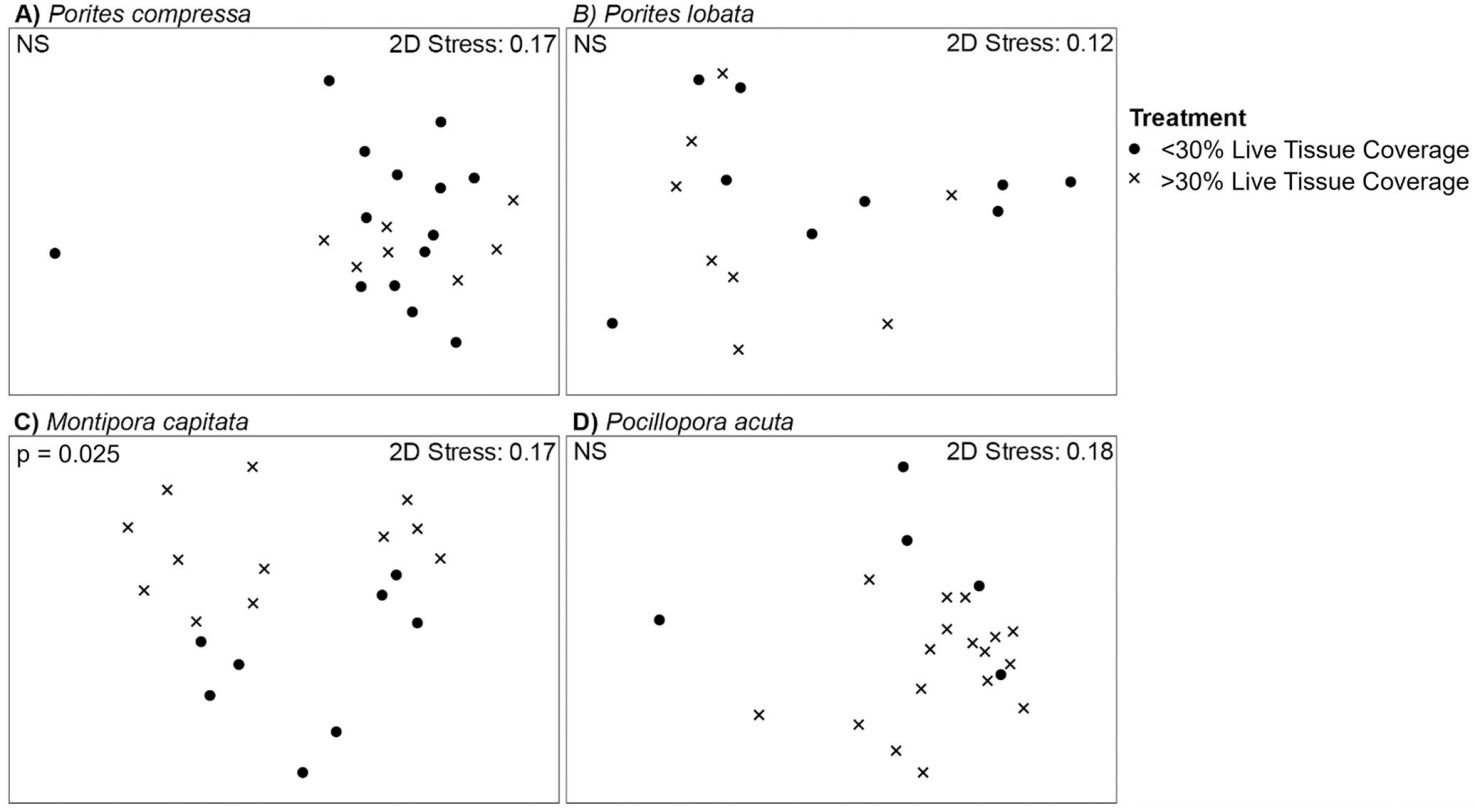
NMDS plots of coral-associated microbial community composition of A) *Porites compressa*, B) *Porites lobata*, C) *Montipora capitata*, and D) *Pocillopora acuta* in the control categorized by greater than 30% live tissue coverage (closed circles) or less than 30% live tissue coverage (X) of their genetic counterparts in the future ocean treatment (Illustrated in Fig 1B). Significant differences detected by PERMANOVA analyses (p < 0.05) are indicated in the top left corner (NS = no significant difference) with corresponding statistical details in S11 Table in S1 File.

Coral genets within the control were compared based on the fate (i.e., greater than or less than 30% live tissue coverage) of their corresponding ramet from the same genet in the future ocean treatment (illustrated in Fig 1B). Alpha and beta diversity of the microbial communities associated with *P*. *compressa*, *P*. *lobata*, and *P*. *acuta* genets with >30% live tissue coverage in the future ocean treatment did not significantly differ from those with <30% live tissue (S13 Table in S1 File), but beta diversity did differ in *M*. *capitata* (Fig 5; S14 Table in S1 File). Surviving genets of *M*. *capitata* with >30% live tissue had a high relative abundance of a specific *Endozoicomonas* sp. OTU (mean of 18.5%, JOKG01000007.7100.8645) and no presence of an OTU in the Rickettsiales (mean of 0.0%, KC682789.1.1464) compared to the genets that had <30% live tissue in the future ocean treatment (mean of 0.67% and 5.38%, respectively) (S15 Table in S1 File).

## Discussion

Following 22-months of chronic ocean warming and acidification, surviving Hawaiian corals exhibited one of two possible response patterns: (1) microbial communities shifted in response to the treatments, potentially suggestive of acclimatization or (2) microbial communities did not change. The first strategy was observed in both *Porites* species and was associated with high survivorship compared to *M*. *capitata* and *P*. *acuta*, which both exhibited the second strategy (Fig 2). There is lower survivorship of corals in the second category, but it is not uniform: there is also variation in performance among individuals with differing, but unchanged, microbial communities. For example, surviving genets of *M*. *capitata* had different, presumably more resistant communities, than genets that died or had <30% live tissue.

### *Porites compressa* and *Porites lobata*

Our findings suggest that higher survivorship in *P compressa* and *P*. *lobata* under future ocean conditions is associated with acclimatization of their microbial communities. Two lines of evidence lead us to this conclusion. First, on the reef, both coral species host different microbial communities that differ based on their collection location [39]. Following 22 months in the experimental control, no site differences were detected within each species (S3B & S3C Table in S1 File), suggesting that the microbial communities had changed in response to their environment in the mesocosms. Second, the beta diversity of the microbial communities of *P*. *lobata* in the control differed from those in the ocean warming and future ocean treatments, and the communities associated with *P*. *compressa* differed between the control and future ocean treatment (Fig 5A and 5B, S7A, S7B Table in S1 File). These findings suggest that microbial communities associated with *Porites* corals changed in response to their new environmental conditions in the ocean warming and/or future ocean treatments. Short-term studies find that shifts in microbial community composition can be accompanied by a decline in overall coral health [e.g., 35, 41, 45]. However, longer-term translocation and monitoring studies often find that gradual changes in the microbial communities associated with some species are a typical response to environmental change, particularly over multi-annual time scales [e.g., 71, 72] and when exposed to new conditions [e.g., 34]. In this case, after nearly two years of chronic future ocean conditions, the shifts in microbial community composition observed here may represent acclimatization to future ocean conditions by these corals, given that these two species also had the highest survivorship rates among the four species in the experiment (Fig 2) and many showed strong physiological performance under these conditions [53]. Indeed, *Porites* corals are often among the most resistant to bleaching and mortality in the Hawaiian Islands [17, 73, 74], although this can vary among location [13].

The difference in the microbial community composition between the control and the future ocean treatment in both *Porites* corals was largely attributed to a reduction in the relative abundance of the bacterial genus, *Kistimonas* sp., in the order Oceanospirillales (S10 Table in S1 File, S3 Fig). Given that other bacterial genera of the order Oceanospirillales (i.e., *Endozoicomonas*) are known to persist in coral-associated microbial communities at elevated temperatures [e.g., 43], it is unclear why the *Porites* corals had such a marked decrease in relative abundance of *Kistimonas* sp. in the future ocean treatment. The high survivorship of *Porites* corals and the minimal changes in relative abundance of potentially pathogenic bacteria (i.e., *Vibrio* sp.) [75] suggests that relative decreases of *Kistimonas* sp. losses were replaced with other community members more tolerant of the altered environmental conditions.

While metrics of alpha diversity for both Porites corals increased in the future ocean treatment, SIMPER analyses revealed that the greatest contributors to changes in community composition came from decreases in the relative abundance of several community members (S10A, S10B, Table in S1 File), including the aforementioned OTU associated with the genus, Kistimonas. However, in Porites compressa, one notable relative increase came from Candidatus Amoebophilus (JQ515688.1.1518), which had a threefold increase in relative abundance in the future ocean treatment compared to the control (S10A Table in S1 File). This bacterial genus is hypothesized to interact with intracellular protists, such as Symbiodiniaceae [76], suggesting a potential change to the relationship between the bacterial community and the Symbiodiniaceae in *Porites compressa* under future ocean conditions that could be investigated further.

Interestingly, within the control and future ocean treatments there was no significant correlation between the microbial communities and the overall physiological profile of the corals. McLachlan et al. [53] found that these same *Porites* corals in the future ocean treatment were able to maintain positive calcification, as well as total biomass and lipid levels. Given the apparent shift in microbial community composition and maintenance of key physiological parameters, we hypothesize that the flexibility of the microbial community supported the maintenance of overall *Porites* coral holobiont health, rather than correlating with changes in coral physiology. However, additional sampling timepoints and potentially functional profiling of the microbial communities throughout the experiment would be needed to test this hypothesis. Finally, Rocha de Souza [77] found that the *Porites* corals in this same experiment were dominated by the Symbiodiniaceae genus, *Cladocopium*, and this remained largely consistent regardless of treatment. With the maintenance of most physiological parameters and the consistency of the Symbiodiniaceae genus in *Porites* corals, the shifts of these *Porites*-associated microbial communities by the end of the experiment provides a potential mechanism for supporting their tolerance to future ocean conditions.

### *Montipora capitata* and *Pocillopora acuta*

In contrast to the two *Porites* species, we find that lower survivorship in *M*. *capitata* and *P*. *acuta* under future ocean conditions is associated with a lack of change in their microbial community composition in the ocean warming and future ocean treatments. Both *M*. *capitata* and *P*. *acuta* ramets within the control maintained distinct microbial communities based on their collection site after almost two years in the mesocosm (S3D, S3E Table in S1 File). This stability, even under common environmental conditions of the control mesocosms, suggests that the microbial communities of these corals are unresponsive to environmental changes, relative to the *Porites* corals. For *P*. *acuta* specifically, this pattern supports previous findings of stability in associated microbial communities under heat stress, with greater differences in community composition being related to reef location [78, 79]. However, in *M*. *capitata*, genets that died or had less than 30% live tissue coverage after 22 months in the future ocean treatment had microbial communities that differed from genets that survived with greater than 30% live tissue (Fig 4C, S14 Table in S1 File). These findings suggest that the microbial communities of surviving *M*. *capitata* do not respond to environmental conditions, but that some genets already have microbial communities that can persist in future ocean conditions, which may affect coral holobiont performance and survival under future ocean conditions.

Our finding of lower survivorship in coral species with a seemingly stable microbiome contrasts with findings showing that stable microbial community composition is associated with better physiological health in *Turbinaria reniformis* [35]. However, this previous study was conducted over only a few weeks, as compared to the 22-month timeframe of this experiment. Rather, our results suggest that individual *M*. *capitata* corals either have microbial communities associated with tolerance to future ocean conditions or they do not. Since survivorship was dominated by *M*. *capitata* genets sourced from the naturally warmer and more acidic Kāne‘ohe Bay sites of Moku o Lo‘e and Sampan [17, 18, 39, 80], the findings further indicate that environmental history of these coral species may have already conditioned some genets to host microbial communities that promote resistance to future ocean conditions. Indeed, *M*. *capitata* genets that survived the future ocean treatment had a greater relative abundance of several *Endozoicomonas* sp. OTUs when compared with those genets that died or had little remaining live tissue (S15 Table in S1 File).

Greater relative abundance of the *Endozoicomonas* sp. may be an indicator of improved resistance to future ocean conditions in *M*. *capitata*. These bacteria are known to have important roles in nutrient cycling, host health, and control over the microbial community composition [81]. In parallel to our findings here for *M*. *capitata*, *s*everal other studies have also found *Endozoicomonas* bacteria to be associated with coral health and/or resilience [e.g., 75, 79, 82]. Interestingly, this contrasts with our findings in the *Porites* corals which hosted substantially lower abundances of Oceanospirillales bacteria (*Kistomonas* sp. and *Endozoicomonas* sp.) in the future ocean treatment (S10 Table in S1 File & S3 Fig). This indicates that there is not a single universal microbial group that can serve as an indicator for coral tolerance and resilience to predicted future ocean conditions. An additional factor influencing the survival of certain *M*. *capitata* genets may be the composition of their Symbiodiniaceae communities, such that survivors were more commonly dominated by the thermotolerant genus *Durisdinium* after 22 months in the elevated temperature treatments [77].

The low survivorship of *P*. *acuta* suggests that the microbial community associated with this species was also somewhat inflexible, but unlike *M*. *capitata*, the microbial community of *P*. *acuta* did not predict survivorship under future ocean conditions (S14 Table in S1 File). The low survivorship is consistent with findings from additional studies of Hawaiian *P*. *acuta*, which hypothesize that the host-Symbiodiniaceae relationship for this coral species is sensitive to environmental stresses like heat and nutrient enrichment [83, 84]. This species of coral is often fast-growing, reproduces at a young age, and shows high rates of recruitment, lending to its characterization as a “weedy coral” [85]. Though fewer genets of *P*. *acuta* survived chronic future ocean conditions for 22 months as compared to other species (Fig 2), those survivors may proliferate quickly [e.g., 86]. The high recruitment rates of new *P*. *acuta* colonies into all mesocosm tanks, irrespective of treatment [51], suggests that the few resilient genets that persisted could rapidly fill the ecological niches left by the large number of lost genets, though potentially at the cost of reduced genetic diversity.

### Implications

All four species of Hawaiian corals had surviving genets after 22 months of chronic ocean warming and acidification stress. In support of our hypothesis, our findings indicate that *Porites* coral microbial communities appear to acclimatize to future ocean conditions, which is associated with higher survivorship among these corals as compared to the other species. In contrast, though *M*. *capitata* hosts relatively inflexible microbial communities, the microbial communities of some genets may already be more tolerant of warmer conditions. While the former strategy may be more successful and *Porites* corals could become more abundant on coral reefs of the future, all four species include individuals that survived this chronic exposure to future ocean conditions and could potentially persist on reefs over the coming decades. Further, species-specific patterns in the responses of coral microbial communities to future ocean conditions may provide some strategies for coral restoration. For example, microbial community composition would not be an important factor in the selection of *Porites* corals for transplantation to other sites or for restoration. Whereas, *M*. *capitata* ramets harvested from warmer sites, such as Kāne‘ohe Bay, are more likely to have microbial communities that are pre-selected for survival under future ocean conditions and may be better targets for restoration than corals sourced from elsewhere around O‘ahu.

## Supporting information

S1 Fig Coral collection sites surrounding O’ahu, HI, USA. MoL = Moku o Lo‘e. Specific coordinates of each site are listed in Table 1.
(TIF)

S2 Fig Rarefaction curves for each sample in the study (N = 239) at a step of 100 reads. (TIF)

S3 Fig Mean relative abundances of the most common microbial Orders associated with surviving genets of each coral species in the control condition and the future ocean treatment (illustrated in Fig 1A) in (A) *Porites compressa*, (B) *Porites lobata*, (C) *Montipora capitata*, and (D) *Pocillopora acuta*. Only Orders with a relative abundance greater than 2% in at least one coral species are represented individually.
(TIF)

S1 File Supporting information tables. (DOCX)
