# Supplementary figures and images for: Long-term coral microbial community acclimatization is associated with coral survival in a changing climate
Source: PLoS One. 2023 Sep 22;18(9):e0291503. doi: 10.1371/journal.pone.0291503 (PMC10516427; doi:10.1371/journal.pone.0291503)

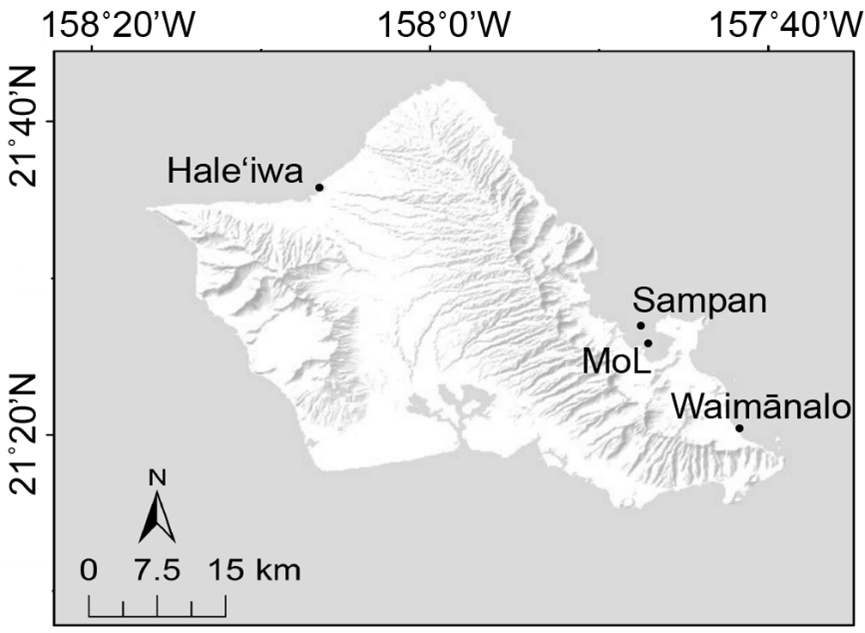

Supplement: S1 Fig — MoL = Moku o Lo‘e. Specific coordinates of each site are listed in Table 1. (TIF) [file pone.0291503.s001.tif]

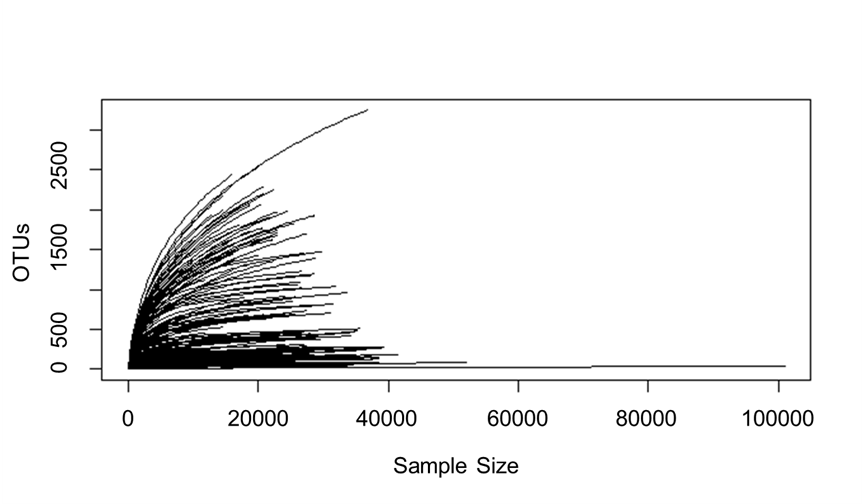

Supplement: S2 Fig — (TIF) [file pone.0291503.s002.tif]

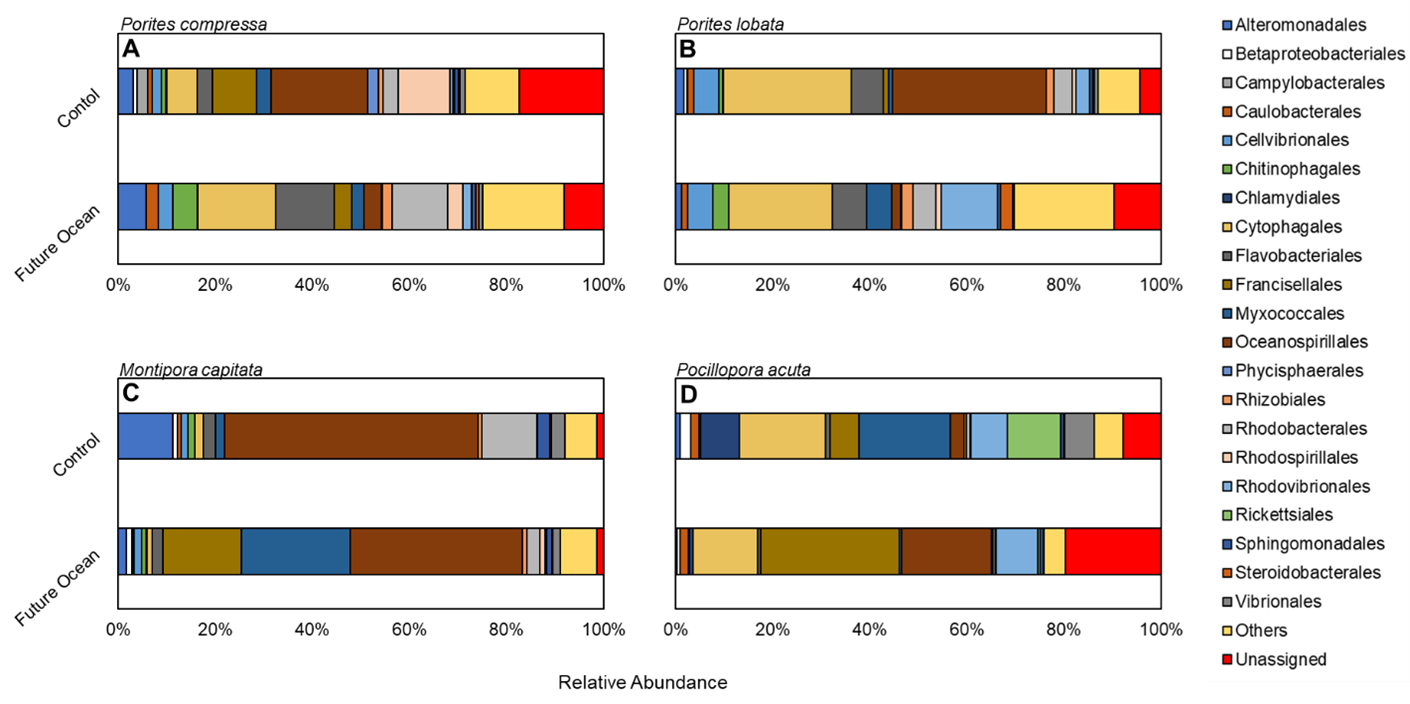

Supplement: S3 Fig — Only Orders with a relative abundance greater than 2% in at least one coral species are represented individually. (TIF) [file pone.0291503.s003.tif]
