# Supplementary material for: Long-term coral microbial community acclimatization is associated with coral survival in a changing climate
Source: PLoS One. 2023 Sep 22;18(9):e0291503. doi: 10.1371/journal.pone.0291503 (PMC10516427; doi:10.1371/journal.pone.0291503)
Supplement: S1 File — (DOCX) [file pone.0291503.s004.docx]

**Supporting Information:** Long-term coral microbial community acclimatization is associated with coral survival in a changing climate

James T. Price^1*^, Rowan H. McLachlan^1,2^, Christopher P. Jury^3^, Robert J. Toonen^3^, Michael J. Wilkins^4^, Andréa G. Grottoli^1*^

**Table S1** Alpha diversity metrics for the microbial communities associated with each coral species in the control. No significant differences (p < 0.05) were found among species for any alpha diversity metric.

|  | | | | | | |  |  |  |  |
| --- | --- | --- | --- | --- | --- | --- | --- | --- | --- | --- |
| Species | Observed OTUs | | Chao1 | | Shannon | | | Faith's PD | | |
| *P. compressa* (n = 21) | 433.57 ± 507.35 | | 594.54 ± 697.82 | | 3.04 ± 1.38 | | | 41.74 ± 34.80 | | |
| *P. lobata* (n = 17) | 657.47 ± 743.58 | | 397.29 ± 375.56 | | 3.31 ± 1.85 | | | 31.06 ± 21.60 | | |
| *M. capitata* (n = 20) | 451.40 ± 529.98 | | 570.11 ± 668.89 | | 3.56 ± 1.29 | | | 39.87 ± 39.50 | | |
| *P. acuta* (n = 22) | 255.82 ± 222.25 | | 349.85 ± 323.02 | | 2.75 ± 1.01 | | | 28.37 ± 18.44 | | |
|  | |  | |  | |  | | |  |  |

**Table S2** Two-way PERMANOVA of the coral-associated microbial communities of each species and collection site (nested within species). Post-hoc pairwise comparisons are shown in Table S3. df = degrees of freedom.

|  |  |  |  |  |
| --- | --- | --- | --- | --- |
| Groups | df | Pseudo-F | P-value | Permutations |
| Species | 3 | 2.666 | **0.001** | 9861 |
| Site (nested within species) | 11 | 1.138 | **0.034** | 9612 |

**Table S3** PERMANOVA post-hoc pairwise statistics comparing A) the coral-associated microbial communities of each species in the control. PERMANOVA statistics for comparisons among corals within species in the control based on their collection location in (B) *Porites compressa*, (C) *Porites lobata*, (D) *Montipora capitata*, and (E) *Pocillopora acuta*.

| **A) By Species** |  |  |  |
| --- | --- | --- | --- |
| **Groups** | **t-value** | **P-value** | **Permutations** |
| *Porites compressa* ‒ *Porites lobata* | 1.282 | **0.024** | 999 |
| *Porites compressa* ‒ *Montipora capitata* | 1.578 | **0.001** | 999 |
| *Porites compressa* ‒ *Pocillopora acuta* | 1.893 | **0.001** | 999 |
| *Porites lobata* ‒ *Montipora capitata* | 1.808 | **0.001** | 999 |
| *Porites lobata ‒ Pocillopora acuta* | 1.786 | **0.002** | 999 |
| *Montipora capitata ‒ Pocillopora acuta* | 2.127 | **0.001** | 999 |
|  |  |  |  |
| **B) *Porites compressa*** |  |  |  |
| **Groups** | **t-value** | **P-value** | **Permutations** |
| Hale‘iwa ‒ Moku o Lo‘e | 1.025 | 0.269 | 462 |
| Hale‘iwa ‒ Sampan | 0.988 | 0.524 | 126 |
| Hale‘iwa ‒ Waimānalo | 1.000 | 0.473 | 126 |
| Moku o Lo‘e ‒ Sampan | 0.930 | 0.589 | 462 |
| Moku o Lo‘e ‒ Waimānalo | 0.881 | 0.834 | 462 |
| Sampan ‒ Waimānalo | 1.070 | 0.187 | 126 |
|  |  |  |  |
| **C) *Porites lobata*** |  |  |  |
| **Groups** | **t-value** | **P-value** | **Permutations** |
| Hale‘iwa ‒ Sampan | 0.794 | 0.831 | 462 |
| Hale‘iwa ‒ Waimānalo | 0.772 | 0.949 | 462 |
| Sampan ‒ Waimānalo | 0.821 | 0.841 | 462 |
|  |  |  |  |
| **D) *Montipora capitata*** |  |  |  |
| **Groups** | **t-value** | **P-value** | **Permutations** |
| Hale‘iwa ‒ Moku o Lo‘e | 1.397 | **0.024** | 84 |
| Hale‘iwa ‒ Sampan | 1.552 | **0.017** | 56 |
| Hale‘iwa ‒ Waimānalo | 1.297 | 0.059 | 84 |
| Moku o Lo‘e ‒ Sampan | 1.120 | 0.263 | 462 |
| Moku o Lo‘e ‒ Waimānalo | 1.206 | 0.076 | 462 |
| Sampan ‒ Waimānalo | 1.139 | 0.228 | 462 |
|  |  |  |  |
| **E) *Pocillopora acuta*** |  |  |  |
| **Groups** | **t-value** | **P-value** | **Permutations** |
| Hale‘iwa ‒ Moku o Lo‘e | 1.285 | **0.046** | 210 |
| Hale‘iwa ‒ Sampan | 1.091 | 0.201 | 462 |
| Hale‘iwa ‒ Waimānalo | 1.009 | 0.343 | 462 |
| Moku o Lo‘e ‒ Sampan | 0.981 | 0.490 | 210 |
| Moku o Lo‘e ‒ Waimānalo | 1.291 | **0.047** | 210 |
| Sampan ‒ Waimānalo | 1.088 | 0.266 | 462 |

**Table S4** Alpha diversity metrics for the microbial communities associated with each of the collection sites for each coral species in the control. Letters indicate significant differences (p < 0.05) among the alpha diversity metrics within each species.

| Species | Observed OTUs | Chao1 | Shannon | Faith's PD |
| --- | --- | --- | --- | --- |
| ***Porites compressa*** |  |  |  |  |
| Hale‘iwa | 485.20 ± 492.27 | 667.74 ± 659.58 | 3.66 ± 1.15 | 46.33 ± 32.25 |
| Moku o Lo‘e | 326.33 ± 184.41 | 463.40 ± 214.81 | 3.02 ± 1.34 | 35.80 ± 15.48 |
| Sampan | 775.00 ± 860.74 | 1049.65± 1212.35 | 3.02 ± 2.21 | 64.43 ± 58.33 |
| Waimānalo | 169.20 ± 87.49 | 223.58 ± 93.90 | 2.45 ± 0.37 | 21.58 ± 9.11 |
| ***Porites lobata*** |  |  |  |  |
| Hale‘iwa | 415.00 ± 389.19 | 591.46 ± 480.80 | 3.07 ± 2.05 | 41.36 ± 31.87 |
| Sampan | 862.83 ± 903.56 | 1212.56 ± 1291.86 | 3.46 ± 1.90 | 70.04 ± 59.23 |
| Waimānalo | 654.83 ± 850.07 | 937.24 ± 1230.06 | 3.37 ± 1.97 | 53.04 ± 58.32 |
| ***Montipora capitata*** |  |  |  |  |
| Hale‘iwa | 938.00 ± 439.95^a^ | 1210.17 ± 711.93^a^ | 5.00 ± 0.15^a^ | 81.42 ± 31.90^a^ |
| Moku o Lo‘e | 151.33 ± 150.97^b^ | 210.02 ± 175.247^b^ | 2.77 ± 1.05^b^ | 15.37 ± 11.66^b^ |
| Sampan | 341.20 ± 486.16^a,b^ | 399.53 ± 537.22^a,b^ | 3.06 ± 1.15^a,b^ | 31.10 ± 37.85^a,b^ |
| Waimānalo | 600.00 ± 699.48^a,b^ | 752.21 ± 874.92^a,b^ | 4.04 ± 1.29^a,b^ | 50.90 ± 47.54^a,b^ |
| ***Pocllopora acuta*** |  |  |  |  |
| Hale‘iwa | 168.50 ± 75.27^a,b^ | 207.30 ± 76.46^a,b^ | 2.58 ± 0.63 | 20.79 ± 5.99^a,b^ |
| Moku o Lo‘e | 133.25 ± 38.00^a^ | 178.04 ± 43.40^a^ | 2.17 ± 1.07 | 17.13 ± 1.56^a^ |
| Sampan | 295.00 ± 288.74^a,b^ | 461.18 ± 482.26^a,b^ | 3.10 ± 1.22 | 32.16 ± 23.04^b^ |
| Waimānalo | 385.67 ± 272.62^b^ | 495.63 ± 332.14^b^ | 2.97 ± 1.09 | 39.63 ± 22.83^b^ |

**Table S5** SIMPER statistics output of the top 25% of OTU’s most responsible for significant differences between sites within species as determined by PERMANOVA (See Table S2B-E) in the microbial communities of A) *Montipora capitata*, and B) *Pocillopora acuta.*  MoL = Moku o Lo‘e

| **A) *Montipora capitata*** |  |  |  |  |  |  |
| --- | --- | --- | --- | --- | --- | --- |
| **Sites** | **OTU ID** | **Genus** | **Average Abundance (%)** | **Average Abundance (%)** | **Average Dissimilarity (%)** | **Cumulative Contribution (%)** |
| Hale‘iwa ‒ MoL |  |  | Hale‘iwa | MoL |  |  |
|  | JOKG01000007.7100 | *Endozoicomonas* sp. | 0.00 | 10.47 | 5.23 | 5.45 |
|  | KC682789.1.1464 | Midichloriaceae_MD3-55 | 0.00 | 8.99 | 4.49 | 10.12 |
|  | FNAP01000038.454.1940 | *Roseospira* sp. | 8.42 | 0.00 | 4.21 | 14.50 |
|  | CP013251.969397.970960 | *Endozoicomonas* sp. | 0.01 | 5.08 | 2.54 | 17.15 |
|  | JN695900.1.1531 | *Methylobacterium radiotolerans* | 0.10 | 4.83 | 2.37 | 19.61 |
|  | JF277164.1.1494 | *Cutibacterium* sp. | 0.00 | 4.49 | 2.24 | 21.94 |
|  | New.ReferenceOTU468 | *Endozoicomonas* sp. | 0.02 | 4.27 | 2.13 | 24.16 |
|  | JQ515626.1.1464 | Midichloriaceae_MD3-55 | 0.00 | 3.95 | 1.98 | 26.22 |
| Hale‘iwa ‒ Sampan |  |  | Hale‘iwa | Sampan |  |  |
|  | JOKG01000007.7100 | *Endozoicomonas* sp. | 0.00 | 17.12 | 8.56 | 8.88 |
|  | FNAP01000038.454.1940 | *Roseospira* sp. | 8.42 | 0.00 | 4.21 | 13.25 |
|  | CP013251.969397.970960 | *Endozoicomonas* sp. | 0.01 | 7.52 | 3.76 | 17.15 |
|  | JN624846.1.1450 | *Endozoicomonas* sp. | 0.00 | 7.37 | 3.68 | 20.97 |
|  | New.ReferenceOTU468 | *Endozoicomonas* sp. | 0.00 | 5.70 | 2.85 | 23.93 |
|  | KC169771.1.1506 | *Endozoicomonas* sp. | 0.00 | 4.90 | 2.45 | 26.47 |
| Hale‘iwa ‒ Waimānalo |  |  | Hale‘iwa | Waimānalo |  |  |
|  | FNAP01000038.454.1940 | *Roseospira* sp. | 8.42 | 0.00 | 4.21 | 4.72 |
|  | AB128872.1.1589 | *Sphingobium* sp. | 0.00 | 6.61 | 3.31 | 8.43 |
|  | CP000112.1309450.1310979 | Desulfovibrionales_Unclassified | 0.00 | 6.30 | 3.15 | 11.96 |
|  | New.ReferenceOTU525 | Myxococcales_Unclassified | 0.00 | 6.17 | 3.09 | 15.43 |
|  | JN624846.1.1450 | *Endozoicomonas* sp. | 0.00 | 5.05 | 2.53 | 18.26 |
|  | EU567031.1.1505 | *Shewanella* | 3.65 | 0.02 | 1.82 | 20.31 |
|  | DQ395503.1.1453 | Rhodobacteraceae_Unclassified | 2.62 | 2.03 | 1.76 | 22.28 |
|  | AJKS02000002.128589.130106 | Halomonas *sp.* | 0.01 | 3.06 | 1.52 | 23.98 |
|  | KU996359.1.1470 | *Labrenzia* sp. | 2.64 | 0.00 | 1.32 | 25.46 |
| MoL ‒ Waimānalo |  |  | MoL | Waimānalo |  |  |
|  | JOKG01000007.7100 | *Endozoicomonas* sp. | 10.47 | 1.20 | 5.61 | 6.09 |
|  | KC682789.1.1464 | Midichloriaceae_MD3-55 | 8.99 | 1.77 | 4.69 | 11.18 |
|  | New.ReferenceOTU525 | Myxococcales_Unclassified | 1.86 | 6.17 | 3.67 | 15.16 |
|  | AB128872.1.1589 | *Sphingobium* sp. | 0.00 | 6.61 | 3.30 | 18.75 |
|  | JN624846.1.1450 | *Endozoicomonas* sp. | 3.92 | 5.05 | 3.24 | 22.27 |
|  | CP000112.1309450.1310979 | Desulfovibrionales_Unclassified | 0 | 6.30 | 3.15 | 25.69 |
| Sampan ‒ Waimānalo |  |  | Sampan | Waimānalo |  |  |
|  | JOKG01000007.7100 | *Endozoicomonas* sp. | 17.12 | 1.20 | 8.46 | 9.65 |
|  | JN624846.1.1450 | *Endozoicomonas* sp. | 7.37 | 5.05 | 3.80 | 13.99 |
|  | CP013251.969397.970960 | *Endozoicomonas* sp. | 7.52 | 0.49 | 3.77 | 18.28 |
|  | New.ReferenceOTU525 | Myxococcales_Unclassified | 1.82 | 6.17 | 3.66 | 22.45 |
|  | AB128872.1.1589 | *Sphingobium* sp. | 3.05 | 6.61 | 3.32 | 26.23 |
| MoL ‒ Sampan |  |  | MoL | Sampan |  |  |
|  | JOKG01000007.7100 | *Endozoicomonas* sp. | 10.47 | 17.12 | 10.83 | 12.11 |
|  | CP013251.969397.970960 | *Endozoicomonas* sp. | 5.08 | 7.52 | 5.02 | 17.72 |
|  | KC682789.1.1464 | Midichloriaceae_MD3-55 | 8.99 | 0.01 | 4.49 | 22.74 |
|  | JN624846.1.1450 | *Endozoicomonas* sp. | 3.92 | 7.37 | 4.05 | 27.27 |
|  |  |  |  |  |  |  |
| **B) *Pocillopora acuta*** |  |  |  |  |  |  |
| **Sites** |  | **Genus** | **Average Abundance (%)** | **Average Abundance (%)** | **Average Dissimilarity (%)** | **Cumulative Contribution (%)** |
| Hale‘iwa ‒ MoL |  |  | Hale‘iwa | MoL |  |  |
|  | JQ515688.1.1518 | *Candidatus* Amoebophilus sp. | 26.86 | 7.07 | 11.51 | 12.88 |
|  | New.ReferenceOTU55 | Myxococcales_P3OB-42_Unclassified | 0.00 | 22.93 | 11.47 | 25.72 |
| Hale‘iwa ‒ Sampan |  |  | Hale‘iwa | Sampan |  |  |
|  | JQ515688.1.1518 | *Candidatus* Amoebophilus sp. | 26.86 | 23.95 | 14.92 | 17.35 |
|  | AP014635.274077.275609 | *Vibrio* sp. | 5.96 | 0.08 | 2.98 | 20.82 |
|  | New.ReferenceOTU474 | Simkaniaceae_Unclassified | 0.00 | 5.52 | 2.76 | 24.03 |
|  | HM768626.1.1400 | *Endozoicomonas* sp. | 5.48 | 0.00 | 2.74 | 27.22 |
| Hale‘iwa ‒ Waimānalo |  |  | Hale‘iwa | Waimānalo |  |  |
|  | JQ515688.1.1518 | *Candidatus* Amoebophilus sp. | 26.86 | 26.59 | 10.95 | 14.17 |
|  | New.ReferenceOTU300 | *Francisella* sp. | 0.42 | 11.80 | 5.86 | 21.76 |
|  | JN624846.1.1450 | *Endozoicomonas* sp. | 2.21 | 7.13 | 3.60 | 26.42 |
| MoL ‒ Waimānalo |  |  | MoL | Waimānalo |  |  |
|  | New.ReferenceOTU55 | Myxococcales_P3OB-42_Unclassified | 22.93 | 0.08 | 11.49 | 13.72 |
|  | JQ515688.1.1518 | *Candidatus* Amoebophilus sp. | 7.07 | 26.59 | 10.46 | 26.21 |
| Sampan ‒ Waimānalo |  |  | Sampan | Waimānalo |  |  |
|  | JQ515688.1.1518 | *Candidatus* Amoebophilus sp. | 23.95 | 26.59 | 14.44 | 17.67 |
|  | New.ReferenceOTU300 | *Francisella* sp. | 3.61 | 11.80 | 5.85 | 24.82 |
|  | JN624846.1.1450 | *Endozoicomonas* sp. | 0.03 | 7.13 | 3.56 | 29.18 |
| MoL ‒ Sampan |  |  | MoL | Sampan |  |  |
|  | JQ515688.1.1518 | *Candidatus* Amoebophilus sp. | 7.07 | 23.95 | 12.07 | 13.84 |
|  | New.ReferenceOTU55 | Myxococcales_P3OB-42_Unclassified | 22.93 | 0.00 | 11.47 | 26.99 |

**Table S6** PERMANOVA statistics comparing the overall physiological profile of each species in the control.

| **A)** Group | df | Pseudo-F | P-value | Permutations |
| --- | --- | --- | --- | --- |
| Species | 2 | 9.558 | 0.001 | 9917 |
|  |  |  |  |  |
| **B)** Species |  | t-value | P-value | Permutations |
| *P. compressa – P. lobata* |  | 1.564 | **0.015** | 9944 |
| *P. compressa – M. capitata* |  | 3.692 | **0.001** | 9948 |
| *P. lobata – M. capitata* |  | 3.554 | **0.001** | 9935 |

**Table S7** Global BEST statistics testing the relationship between the overall physiological profile and the microbial community associated with coral ramets in the control.

| Species | Rho-statistic | P-value | Permutations |
| --- | --- | --- | --- |
| *Porites compressa* | 0.136 | 0.890 | 99 |
| *Porites lobata* | 0.245 | 0.260 | 99 |
| *Montipora capitata* | 0.240 | 0.110 | 99 |

**Table S8** Alpha diversity metrics for the microbial communities associated with the surviving ramets of each coral species in the (A) ocean acidification treatment, (B) ocean warming treatment, and (C) future ocean treatment compared to the ramets of the same genets in the control (illustrated in Fig 1A). Alpha diversity values that significantly differ are annotated with different letter within each alpha diversity metric comparison within each species.

| **A) Control and Acidification Treatment** | | | | | | | | | | | | | | | | | | | | | | | | | | |  |  |  |  |  |  |
| --- | --- | --- | --- | --- | --- | --- | --- | --- | --- | --- | --- | --- | --- | --- | --- | --- | --- | --- | --- | --- | --- | --- | --- | --- | --- | --- | --- | --- | --- | --- | --- | --- |
| Factor | Observed OTUs | | | Chao1 | | | | | | | Shannon | | | | | | | Faith's PD | | | | | | | | |  |  |  |  |  |  |
| ***P. compressa*** |  |  | |  | | | |  | | |  | | |  | | | |  | | |  | | | | | |  |  |  |  |  |  |
| Control (n = 17) | 419.29 ± 508.51 | | | 566.86 ± 706.11 | | | | | | | 3.10 ± 1.34 | | | | | | | 40.80 ± 35.12 | | | | | | | | |  |  |  |  |  |  |
| Acidification (n = 17) | 535.82 ± 533.38 | | | 707.40 ± 698.73 | | | | | | | 3.43 ± 1.49 | | | | | | | 49.18 ± 38.62 | | | | | | | | |  |  |  |  |  |  |
| ***P. lobata*** |  | | |  | | | | | | |  | | | | | | |  | | | | | | | | |  |  |  |  |  |  |
| Control (n = 17) | 657.47 ± 743.58 | | | 932.71 ± 1057.32 | | | | | | | 3.31 ± 1.85 | | | | | | | 55.61 ± 50.57 | | | | | | | | |  |  |  |  |  |  |
| Acidification (n = 17) | 770.53 ± 832.33 | | | 1045.69 ± 1135.98 | | | | | | | 3.94 ± 1.88 | | | | | | | 63.81 ± 55.81 | | | | | | | | |  |  |  |  |  |  |
| ***M. capitata*** |  | | |  | | | | | | |  | | | | | | |  | | | | | | | | |  |  |  |  |  |  |
| Control (n = 19) | 468.58 ± 538.75 | | | 589.85 ± 681.20 | | | | | | | 3.54 ± 1.33 | | | | | | | 41.25 ± 40.08 | | | | | | | | |  |  |  |  |  |  |
| Acidification (n = 19) | 318.37 ± 411.24 | | | 404.56 ± 517.56 | | | | | | | 2.98 ± 1.61 | | | | | | | 31.48 ± 32.11 | | | | | | | | |  |  |  |  |  |  |
| ***P. acuta*** |  | | |  | | | | | | |  | | | | | | |  | | | | | | | | |  |  |  |  |  |  |
| Control (n = 19) | 228.37 ± 186.60 | | | 299.16 ± 231.50 | | | | | | | 2.52 ± 0.85 | | | | | | | 26.07 ± 15.73 | | | | | | | | |  |  |  |  |  |  |
| Acidification (n = 19) | 206.63 ± 134.03 | | | 270.60 ± 157.81 | | | | | | | 2.74 ± 0.91 | | | | | | | 23.84 ± 12.05 | | | | | | | | |  |  |  |  |  |  |
| **B) Control and Warming Treatment** | | | | | | | | | | | | | | |  |  |  |  |  |  |  |  |  |  |  |  |  |  |  |  |  |  |
| Factor | Observed OTUs | | | Chao1 | | | | | | Shannon | | | | | | | Faith's PD | | | | | | | |  |  |  |  |  |  |  |  |
| ***P. compressa*** |  | | |  | | |  | | | | | |  | | | | | |  | | | |  |  | | | |  | |  |  |  |
| Control (n = 12) | 458.58 ± 596.83 | | | 652.63 ± 822.08 | | | | | | | 2.77 ± 1.58 | | | | | | | 42.66 ± 40.94 | | | | | | | | |  |  |  |  |  |  |
| Warming (n = 12) | 599.17 ± 566.27 | | | 777.00 ± 713.78 | | | | | | | 3.79 ± 1.44 | | | | | | | 53.72 ± 39.40 | | | | | | | | |  |  |  |  |  |  |
| ***P. lobata*** |  | | |  | | | | | | |  | | | | | | |  | | | | | | | | |  |  |  |  |  |  |
| Control (n = 12) | 662.75 ± 836.86 | | | 948.06 ± 1188.91 | | | | | | | 3.14 ± 2.02 | | | | | | | 54.36 ± 57.19 | | | | | | | | |  |  |  |  |  |  |
| Warming (n = 12) | 1091.08 ± 928.42 | | | 1579.16 ± 1387.53 | | | | | | | 4.00 ± 1.95 | | | | | | | 82.91 ± 61.81 | | | | | | | | |  |  |  |  |  |  |
| ***M. capitata*** |  | | |  | | | | | | |  | | | | | | |  | | | | | | | | |  |  |  |  |  |  |
| Control (n = 8) | 415.25 ± 454.36 | | | 487.77 ± 502.02 | | | | | | | 3.19 ± 1.41 | | | | | | | 36.82 ± 34.90 | | | | | | | | |  |  |  |  |  |  |
| Warming (n = 8) | 194.25 ± 246.82 | | | 258.83 ± 272.55 | | | | | | | 1.93 ± 0.98 | | | | | | | 21.86 ± 20.37 | | | | | | | | |  |  |  |  |  |  |
| ***P. acuta*** |  | | |  | | | | | | |  | | | | | | |  | | | | | | | | |  |  |  |  |  |  |
| Control (n = 7) | 162.00 ± 36.46 | | | 238.13 ± 91.14 | | | | | | | 2.78 ± 0.76 | | | | | | | 21.15 ± 3.41 | | | | | | | | |  |  |  |  |  |  |
| Warming (n = 7) | 283.43 ± 308.54 | | | 349.17 ± 362.45 | | | | | | | 1.96 ± 1.04 | | | | | | | 30.56 ± 27.57 | | | | | | | | |  |  |  |  |  |  |
|  | | |  | | |  | | |  | | |  | | |  |  | | | |  | |  |  |  |  |  |  |  |  |  |  |  |
| **C) Control and Future Ocean Treatment** | | | | | | | | | | | | | | |  |  |  |  |  |  |  |  |  |  |  |  |  |  |  |  |  |  |
| Factor | Observed OTUs | | | | Chao1 | | | | | | Shannon | | | | | | | Faith's PD | | | | | | | |  |  |  |  |  |  |  |
| ***P. compressa*** |  | | | |  | | | | | |  | | | | | | |  | | | | | | | | |  | |  | |  |  |
| Control (n = 14) | 446.00 ± 558.03^a^ | | | | 614.17 ± 767.98^a^ | | | | | | 2.84 ± 1.52^a^ | | | | | | | 42.58 ± 38.61^a^ | | | | | | | | |  |  |  |  |  |  |
| Future Ocean (n = 14) | 947.21 ± 690.41^b^ | | | | 1366.74 ± 1034.02^b^ | | | | | | 4.01 ± 1.47^b^ | | | | | | | 78.26 ± 46.47^b^ | | | | | | | | |  |  |  |  |  |  |
| ***P. lobata*** |  | | | |  | | | | | |  | | | | | | |  | | | | | | | | |  |  |  |  |  |  |
| Control (n = 9) | 550.33 ± 836.48^a^ | | | | 795.85 ± 1181.49^a^ | | | | | | 2.74 ± 1.84^a^ | | | | | | | 46.20 ± 56.76^a^ | | | | | | | | |  |  |  |  |  |  |
| Future Ocean (n = 9) | 1822.56 ± 785.10^b^ | | | | 2629.62 ± 1099.81^b^ | | | | | | 5.34 ± 0.73^b^ | | | | | | | 132.21 ± 44.16^b^ | | | | | | | | |  |  |  |  |  |  |
| ***M. capitata*** |  | | | |  | | | | | |  | | | | | | |  | | | | | | | | |  |  |  |  |  |  |
| Control (n = 7) | 303.86 ± 353.61 | | | | 366.16 ± 394.95 | | | | | | 2.94 ± 1.32 | | | | | | | 28.21 ± 27.00 | | | | | | | | |  |  |  |  |  |  |
| Future Ocean (n = 7) | 116.86 ± 67.62 | | | | 161.34 ± 82.24 | | | | | | 2.12 ± 1.52 | | | | | | | 15.54 ± 7.80 | | | | | | | | |  |  |  |  |  |  |
| ***P. acuta*** |  | | | |  | | | | | |  | | | | | | |  | | | | | | | | |  |  |  |  |  |  |
| Control (n = 5) | 147.60 ± 32.25 | | | | 192.00 ± 34.51 | | | | | | 2.26 ± 1.06 | | | | | | | 19.67 ± 2.60 | | | | | | | | |  |  |  |  |  |  |
| Future Ocean (n = 5) | 123.60 ± 48.85 | | | | 167.24 ± 71.51 | | | | | | 1.53 ± 0.94 | | | | | | | 18.25 ± 6.61 | | | | | | | | |  |  |  |  |  |  |

**Table S9** PERMANOVA statistics comparing the microbial communities associated with the surviving ramets of each species in the treatments compared to the ramets of the same genets in the control for (A) *Porites compressa* (B) *Porites lobata*, (C) *Montipora capitata*, (D) *Pocillopora acuta* (illustrated in Fig 1A).

| **A) *Porites comressa*** |  | |  | |  | |
| --- | --- | --- | --- | --- | --- | --- |
| **Groups** | | **F-value** | | **P-value** | | **Permutations** |
| Control ‒ Acidification (N = 17) | | 0.735 | | 0.935 | | 9853 |
| Control ‒ Warming (N = 12) | | 1.305 | | 0.077 | | 9835 |
| Control ‒ Future Ocean (N = 14) | | 1.694 | | **0.001** | | 9842 |
|  | |  | |  | |  |
| **B) *Porites lobata*** | |  | |  | |  |
| **Groups** | | **F-value** | | **P-value** | | **Permutations** |
| Control ‒ Acidification (N = 17) | | 0.834 | | 0.647 | | 9907 |
| Control ‒ Warming (N = 12) | | 2.019 | | **0.030** | | 9861 |
| Control ‒ Future Ocean (N = 9) | | 2.602 | | **0.002** | | 8113 |
|  | |  | |  | |  |
| **C) *Montipora capitata*** | |  | |  | |  |
| **Groups** | | **F-value** | | **P-value** | | **Permutations** |
| Control ‒ Acidification (N = 19) | | 1.126 | | 0.294 | | 9904 |
| Control ‒ Warming (N = 8) | | 1.547 | | 0.137 | | 5028 |
| Control ‒ Future Ocean (N = 7) | | 1.220 | | 0.285 | | 1706 |
|  | |  | |  | |  |
| **D) *Pocillopora acuta*** | |  | |  | |  |
| **Groups** | | **F-value** | | **P-value** | | **Permutations** |
| Control ‒ Acidification (N = 19) | | 0.981 | | 0.420 | | 9874 |
| Control ‒ Warming (N = 7) | | 1.518 | | 0.115 | | 1708 |
| Control ‒ Future Ocean (N = 5) | | 0.729 | | 0.727 | | 126 |

**Table S10** SIMPER statistics output of the top 25% of OTU’s most responsible for significant differences in the microbial communities associated with the surviving ramets within the future ocean treatment compared to the ramets of the same genets in the control (illustrated in Fig 1A) for A) *Porites compressa* and B) *Porites lobata*. Only species with significantly different microbial communities (as determined by PERMANOVA in Table S8C) were analyzed.

| **A) *Porites compressa*** |  | |  | | |  | |  | |  |  |  |
| --- | --- | --- | --- | --- | --- | --- | --- | --- | --- | --- | --- | --- |
| Average dissimilarity = 94.82 |  | | | | |  | |  | |  |  |  |
| **OTU** | **Genus** | **Control Average Abundance (%)** | | **Future Ocean Average Abundance (%)** | **Average Dissimilarity (%)** | | **Cumulative Contribution (%)** | |  |  |  |  |
| FNAP01000038.454.1940 | *Roseospira* sp. | 9.36 | | 0.00 | 4.68 | | 4.93 | |  |  |  |  |
| MUIA01000001.189.1735 | *Kistimonas* sp. | 8.51 | | 0.01 | 4.26 | | 9.42 | |  |  |  |  |
| FJ202084.1.1428 | *Caedibacter* sp. | 8.26 | | 0.37 | 4.11 | | 13.76 | |  |  |  |  |
| JQ515688.1.1518 | *Candidatus* Amoebophilus sp. | 1.83 | | 6.08 | 3.51 | | 17.45 | |  |  |  |  |
| New.ReferenceOTU527 | Unassigned | 6.96 | | 0.00 | 3.48 | | 21.12 | |  |  |  |  |
| JR981191.9.1531 | *Endozoicomonas* sp. | 3.57 | | 1.32 | 2.31 | | 23.56 | |  |  |  |  |
| LYSV01000109.29002.30501 | *Fulvivirga* sp. | 0.15 | | 4.41 | 2.27 | | 25.96 | |  |  |  |  |
|  |  | |  | | |  | |  | |  |  |  |
| **B) *Porites lobata*** |  | |  | | |  | |  | |  |  |  |
| Average dissimilarity = 89.52 |  | | | | |  | |  | |  |  |  |
| **OTU** | **Genus** | **Control Average Abundance (%)** | | **Future Ocean Average Abundance (%)** | **Average Dissimilarity (%)** | | **Cumulative Contribution (%)** | |  |  |  |  |
| MUIA01000001.189.1735 | *Kistimonas* sp. | 26.94 | | 0.04 | 13.47 | | 15.04 | |  |  |  |  |
| JQ515688.1.1518 | *Candidatus* Amoebophilus sp. | 17.25 | | 7.72 | 9.88 | | 26.07 | |  |  |  |  |

**Table S11** PERMANOVA statistics comparing the overall physiological profile of coral genets that survived in both the control and future ocean treatment (Illustrated in Fig 1A).

| **Control vs. Future Ocean** |  |  |  |
| --- | --- | --- | --- |
| Species | F-value | P-value | Permutations |
| *Porites compressa* | 2.470 | **0.008** | 9918 |
| *Porites lobata* | 1.454 | 0.185 | 8157 |
| *Montipora capitata* | 1.291 | 0.256 | 1709 |

**Table S12** Global BEST statistics testing the relationship between the overall physiological profile and the microbial community associated with coral genets that survived in both the control and treatment.

| Species | Rho-statistic | P-value | Permutations |
| --- | --- | --- | --- |
| *Porites compressa* | 0.132 | 0.83 | 99 |
| *Porites lobata* | 0.313 | 0.32 | 99 |
| *Montipora capitata* | 0.145 | 0.82 | 99 |

**Table S13** Alpha diversity metrics for the microbial communities associated with coral ramets in the control whose ramets from the same genet in the future ocean treatment that survived with greater than 30% live tissue coverage (LTC) or had less than 30% LTC (Illustrated in Fig 1B). No significant differences (p < 0.05) were found among species for any alpha diversity metric.

| **Survived vs. <30% LTC** | | | | | | | | |
| --- | --- | --- | --- | --- | --- | --- | --- | --- |
|  | Observed OTUs | | Chao1 | | Shannon | | Faith's PD | |
| ***P. compressa*** |  |  |  |  |  |  |  |  |
| Survived (n = 14) | 506.40 ± 586.41 | | 695.43 ± 804.18 | | 2.98 ± 1.56 | | 46.56 ± 40.27 | |
| <30% LTC (n = 7) | 251.50 ± 105.31 | | 342.30 ± 173.64 | | 3.18 ± 0.88 | | 29.68 ± 7.66 | |
| ***P. lobata*** |  | |  | |  | |  | |
| Survived (n = 9) | 550.33 ± 836.48 | | 795.85 ± 1181.49 | | 2.74 ± 1.84 | | 46.20 ± 56.76 | |
| <30% LTC (n = 8) | 778.00 ± 657.85 | | 1086.68 ± 953.27 | | 3.95 ± 1.75 | | 66.19 ± 43.84 | |
| ***M. capitata*** |  | |  | |  | |  | |
| Survived (n = 8) | 415.25 ± 454.36 | | 487.77 ± 502.02 | | 3.19 ± 1.41 | | 36.82 ± 34.90 | |
| <30% LTC (n = 12) | 475.50 ± 593.45 | | 625.00 ± 777.31 | | 3.81 ± 1.20 | | 41.91 ± 43.69 | |
| ***P. acuta*** |  | |  | |  | |  | |
| Survived (n = 5) | 147.60 ± 32.25 | | 192.00 ± 34.51 | | 2.26 ± 1.06 | | 19.67 ± 2.60 | |
| <30% LTC (n = 17) | 287.65 ± 244.62 | | 396.28 ± 355.77 | | 2.90 ± 0.98 | | 30.92 ± 20.35 | |

**Table S14** PERMANOVA statistics comparing the microbial communities associated with coral ramets in the control whose ramets from the same genet in the future ocean treatment survived or died (Illustrated in Fig 1B).

| **Survived vs. Died** |  |  |  |
| --- | --- | --- | --- |
| Species | F-value | P-value | Permutations |
| *Porites compressa* | 1.308 | 0.070 | 9482 |
| *Porites lobata* | 1.124 | 0.296 | 8128 |
| *Montipora capitata* | 1.813 | **0.025** | 9526 |
| *Pocillopora acuta* | 1.141 | 0.268 | 8238 |

**Table S15** SIMPER statistics output of the top 25% of OTU’s most responsible for significant differences in the microbial communities associated with *Montipora capitata* genets in the control condition, based on whether each genet survived or died in the future ocean treatment (illustrated in Fig. 1B). Only species with significantly different microbial communities (as determined by PERMANOVA in Table S13) were analyzed.

| **A) *Montipora capitata*** |  | |  | | |  | |  | |
| --- | --- | --- | --- | --- | --- | --- | --- | --- | --- |
| Average dissimilarity = 91.96 |  | |  | | |  | |  | |
| **OTU** | **Genus** | **Alive: Average Abundance (%)** | | **Dead: Average Abundance (%)** | **Average Dissimilarity (%)** | | **Cumulative Contribution (%)** | |  |
| JOKG01000007.7100.8645 | *Endozoicomonas sp.* | 18.45 | | 0.67 | 9.22 | | 10.03 | |  |
| CP013251.969397.970960 | *Endozoicomonas sp.* | 8.47 | | 0.27 | 4.25 | | 14.65 | |  |
| JN624846.1.1450 | *Endozoicomonas sp.* | 4.67 | | 4.44 | 3.29 | | 18.23 | |  |
| KC682789.1.1464 | Midichloriaceae_MD3-55 | 0.00 | | 5.38 | 2.69 | | 21.16 | |  |
| New.ReferenceOTU468 | *Endozoicomonas sp.* | 3.58 | | 3.20 | 2.65 | | 24.04 | |  |
| New.ReferenceOTU525 | Myxococcales_P3OB-42 | 1.24 | | 3.95 | 2.37 | | 26.62 | |  |
